# Urinary Metabolites Variation After High-Intensity Rowing Training and Potential Biomarker Screening for Exercise-Induced Muscle Damage

**DOI:** 10.3390/ijms26167897

**Published:** 2025-08-15

**Authors:** Jie Wu, Junjie Ding, Ziyue Zhao, Baoguo Wang, Yang Cheng, Yuxian Li, Liming Wang, Shumin Bo, Aiqin Luo, Changyong Zhang, Yue Yi

**Affiliations:** 1 Department of Physical Education, Beijing Institute of Technology, Zhuhai 519000, China; 6220213014@bit.edu.cn; 2 School of Life Science, Beijing Institute of Technology, Beijing 100081, China; 3220242251@bit.edu.cn (J.D.); 3220245350@bit.edu.cn (Z.Z.); 3120221400@bit.edu.cn (B.W.); bitluo@bit.edu.cn (A.L.); 3 Key Laboratory of Molecular Medicine and Biotherapy, Ministry of Industry and Information Technology, Beijing Institute of Technology, Beijing 100081, China; 4 School of Kinesiology and Health, Capital University of Physical Education and Sports, Beijing 100191, China; chengyang2023@cupes.edu.cn (Y.C.); boshumin@cupes.edu.cn (S.B.); 5 School of Medical Technology, Beijing Institute of Technology, Beijing 100081, China; lyxianer@sohu.com (Y.L.); limingw2008@163.com (L.W.); 6 Department of Environmental Science and Engineering, University of Science and Technology of China, Hefei 230026, China; changyongzhang@ustc.edu.cn

**Keywords:** rowing exercise, metabolomics, urinary metabolites, biomarker screening, exercise-induced muscle damage

## Abstract

Exercise-induced muscle damage (EIMD) is the most common health risk in physical exercise. However, instant and non-invasive methods for EIMD prediction have not been reported. Urine is a promising tool for EIMD prediction. However, urinary metabolite variations after EIMD occurrence have not been revealed, and potential biomarkers have not been identified. In this study, eighteen young students without regular exercise habits were recruited to perform high-intensity rowing exercise. EIMD occurrence was determined using blood biochemical analyses and pain assessment. The changes in urinary metabolites were revealed by quasi-targeted metabolomics. Results demonstrated that high-intensity rowing exercise induced EIMD and obviously changed urinary metabolites, including 23 upregulated metabolites and 26 downregulated metabolites. These differential metabolites were related to energy metabolism, exercise performance, and antioxidant metabolism. Among these metabolites, potential urinary biomarkers were identified with high sensitivity and specificity.

## 1. Introduction

Exercise is an effective way of promoting health, preventing chronic diseases, and aiding in post-illness recovery [1]. However, exercise may also induce health risks, the most common of which is exercise-induced muscle damage (EIMD) [2]. EIMD mainly occurs in high-density or unaccustomed training, and the occurrence is strongly related to high-intensity eccentric contraction of skeletal muscles [3]. The eccentric contraction may result in sarcomere overstretching and damage to cellular integrity [3]. Additionally, the eccentric contraction may induce intracellular Ca^2+^ overload and free radical increase, resulting in rapid protein hydrolysis and cell membrane damage [3,4]. The eccentric contraction may also trigger immune responses, leading to the degradation of damaged cells [3]. Delayed onset muscle soreness (DOMS) represents the prevailing phenotype of EIMD, typically appearing 24 h after exercise and resulting in obvious strength loss in a few days [5]. Severe EIMD may result in rhabdomyolysis, posing high risks of renal complications [3]. Therefore, EIMD prediction, especially in the early stage, is crucial for ensuring exercise health and mitigating exercise-related risks.

DOMS is the most common phenotype of EIMD, but it is unsuitable for early diagnosis due to delayed onset. EIMD is typically accompanied by localized edema and swelling, but the two symptoms are also delayed [5]. Thus far, diagnosing EIMD is mainly based on blood biochemical index analyses [6]. Once muscle cells are damaged in EIMD, intracellular creatine kinase (CK) is released, leading to significantly elevated levels of CK in blood [7]. Therefore, CK has been used as an indicator of EIMD. However, increased CK levels are also observed in some other diseases, e.g., infections [8], reducing the specificity for diagnosing EIMD. To increase the accuracy of EIMD diagnosis, oxidative stress and immune response observation were recommended [9]. However, the diagnostic criteria for oxidative stress responses are ambiguous [10], and the use of immune factors also lacks specificity [11]. Most importantly, all of these indexes rely on blood sampling, which requires professional technicians, and may interfere with exercise and even induce secondary infections [12]. Therefore, blood biochemical analysis is unsuitable for EIMD prediction, especially for public fitness.

Compared with blood biochemical analysis, non-invasive methods for EIMD prediction show great prospects. Recent studies have reported some non-invasive assessments of EIMD [12]. According to the obvious decrease in muscle strength, Markus et al. utilized tensiomyography for EIMD assessment [2]. However, the muscle strength decrease occurs approximately 24 h after exercise and is too late for diagnosis. Magnetic resonance imaging was also used to assess EIMD, but obvious changes in macroscopic muscle morphology also appear 24 h after exercise [13]. Compared with the two methods, electromyographic signals are capable of instantly changing after EIMD in terms of muscle excitation and the neuromuscular signal conduction rate [14], but the relationship between electromyographic signals and EIMD is unclear. Furthermore, all of these methods require expensive equipment, limiting the applications in public exercise.

Aside from blood, urine may serve as a tool for EIMD prediction [6,12]. Thus far, some studies have reported changes in urinary metabolites after exercise [15]. For example, Sun et al. compared urinary metabolites before and after an 800 m sprint among 19 athletes, revealing a significant upregulation of 11 metabolites and a significant downregulation of 5 metabolites immediately after exercise [16]. Similarly, other studies have investigated the changes occurring in urinary metabolites after common exercise, e.g., 80 m sprints [17], soccer matches [18], cycling [19], submaximal endurance cycling [20], marathons [21], and resistance training [22]. However, these studies did not analyze EIMD occurrence after exercise, failing to explore the relationship between urinary metabolite changes and EIMD occurrence. Thus far, only a recent study reported changes in urine metabolites following EIMD occurrence [23]. This study identified significant increases in alanine, ethanol, lactate, and asparagine after EIMD onset [23], showing prospects as urinary biomarkers of EIMD. However, these metabolites did not exhibit significant changes immediately after exercise, and only significantly changed 24 h after exercise, indicating the incapability of EIMD prediction. Therefore, the changes in urinary metabolites immediately after exercise should be further revealed, and more efforts are needed to illuminate the potential of urinary metabolites in EIMD prediction.

In this study, urine metabolite variation after high-intensity rowing exercise was revealed, and potential urinary biomarkers for EIMD prediction were identified. First, young participants without regular exercise habits were recruited to perform high-intensity rowing exercise. EIMD occurrence was determined by using blood biochemical analyses. Then, the changes in urinary metabolites were revealed. The relationships between urinary metabolites and blood biochemical indicators were analyzed. Both differential metabolites and enriched metabolic pathways were elucidated. Finally, a discriminative model was established for EIMD prediction, and potential urinary biomarkers for EIMD prediction were identified. The performance of EIMD prediction was compared between single and multiple metabolites.

## 2. Results and Discussion

### 2.1. Biochemical Index and EIMD Analyses

**Figure 1:**
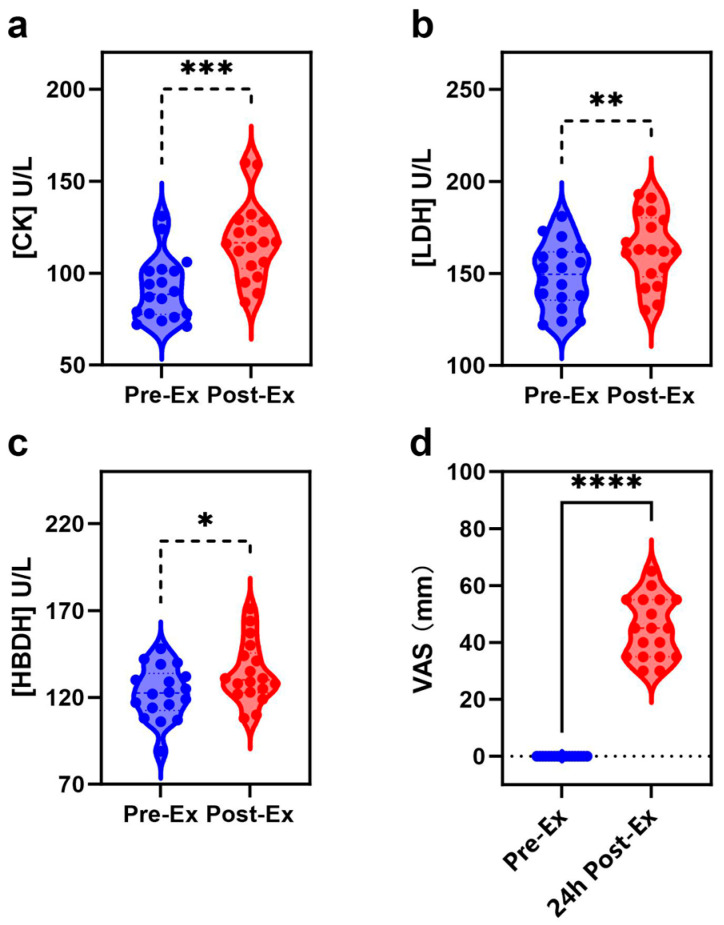
Changes in blood biochemical index of EIMD and pain measurement: (**a**) CK; (**b**) LDH; (**c**) HBDH; and (**d**) VAS. (* represents *p* < 0.0332; ** represents *p* < 0.0021; *** represents *p* < 0.0002; **** represents *p* < 0.0001).

In this study, 18 young male participants without regular exercise habits were asked to perform high-intensity rowing exercise. Figure 1 illustrates the changes in biochemical indexes of EIMD after the exercise. The CK level was 91.4 ± 17.2 U/L pre-exercise, and significantly increased to 116.9 ± 20.6 U/L immediately after the exercise (Figure 1a). CK elevation is due to the disruption of muscle cell integrity and the release of intracellular contents [3], suggesting EIMD occurrence after the exercise. An immediate increase in the CK level after high-intensity exercise and muscle injury has been widely reported in a series of previous studies [24,25,26]. The EIMD occurrence after the exercise is due to three reasons. First, none of the participants had ever performed rowing exercise, i.e., rowing exercise is an unfamiliar training for all of them. Secondly, high-intensity rowing exercise is challenging for participants because none of the participants had regular exercise habits, and the rowing exercise was set at a high resistance level of 30. Thirdly, rowing exercise recruits more than 80% of the muscle groups, mostly through eccentric contractions.

Three other phenomena also indicated that EIMD occurred after high-intensity exercise. First, significant LDH elevation is a typical phenotype of EIMD occurrence [3]. As shown in Figure 1b, LDH significantly increased from 148.6 ± 17.6 to 163.1 ± 18.9 U/L after the exercise. Secondly, HBDH significantly increased from 122.6 ± 15.0 to 134.2 ± 17.4 U/L (Figure 1c). LDH is considered the biomarker of overtraining, and overtraining easily induces EIMD [27]. Most importantly, all participants reported obvious muscle soreness at 24 h after exercise, i.e., DOMS appeared (Figure 1d). Therefore, EIMD occurrence after high-intensity rowing exercise was confirmed.

### 2.2. Metabolite Change Characteristics After High-Intensity Rowing Training

**Figure 2:**
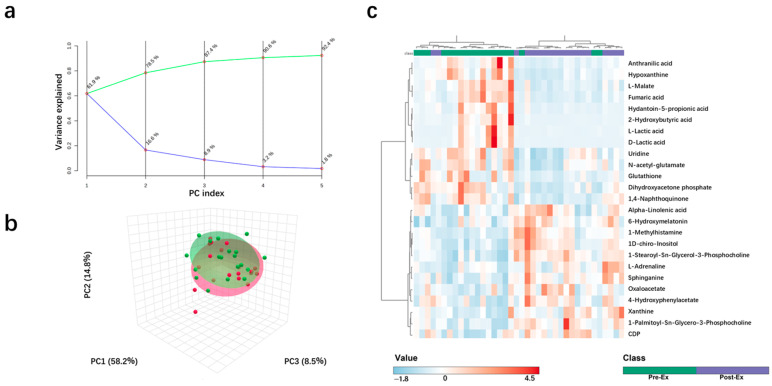
(**a**) The cumulative variance explained by the first N principal components (green line) and proportion of variance explained by each individual principal component (blue line); (**b**) Changes in urinary metabolites after exercise using PCA analysis; (**c**) heatmap and clustering analysis.

**Figure 3:**
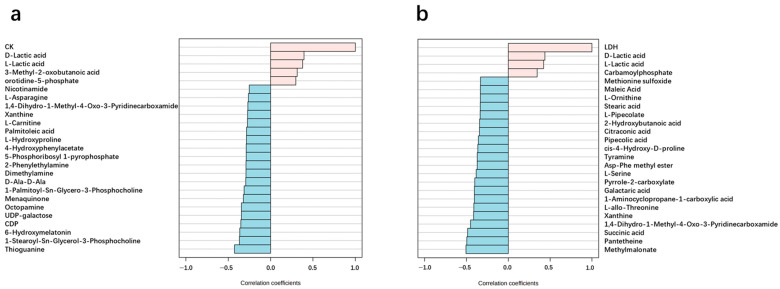
High correlation between urinary metabolites and blood biochemical index: (**a**) CK; (**b**) LDH. (The pink bars represent compounds with a positive correlation with CK or LDH, while the blue bars represent compounds with a negative correlation with CK or LDH).

Urinary metabolites were analyzed using quasi-targeted metabolomics. A total of 742 metabolites were identified among 36 urine samples, including 18 samples collected before exercise and 18 samples collected immediately after the exercise. Among these metabolites, 350 metabolites were annotated by using KEGG and were used for the following analyses. As shown in the PCA, three principal components accounted for more than 85% variance in the urine metabolites. Two groups of urine samples were located at different regions, indicating obvious changes in the urine metabolites after the exercise (Figure 2a,b). Additionally, the changes in the urine metabolites followed a similar trend among all participants. As shown in Figure 2c, samples in the Pre-Ex group basically clustered into one group, while samples in the Post-Ex group basically clustered into another group. Interestingly, some urine metabolites changed with similar trends to CK and LDH. For example, lactic acid has a strong positive correlation with CK and LDH (Figure 3a,b). Both CK and LDH originate from cell disruption and intracellular enzyme release [28]. Therefore, the metabolites strongly correlated with CK, and the LDH variations may also originate from muscle cell rupture, Ca-activated protease products, or reactive oxygen species (ROS)-damaged cell membrane components. Further research is required to elucidate the underlying mechanism.

**Figure 4:**
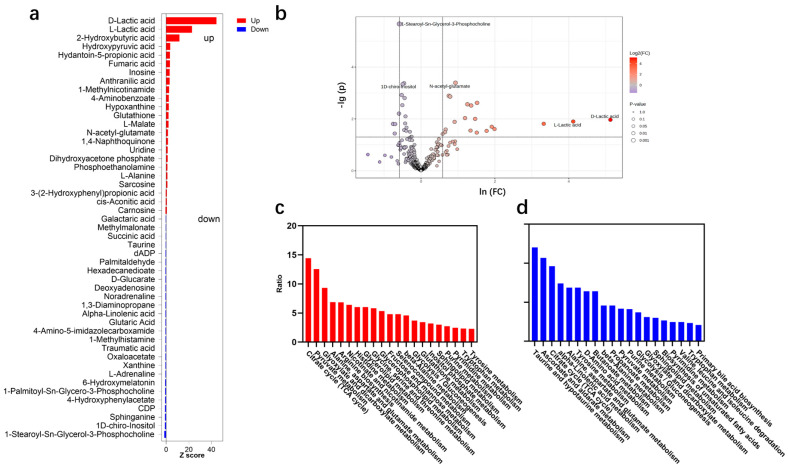
(**a**) Z-scores, (**b**) volcano plot, (**c**) enriched upregulated pathway, and (**d**) enriched downregulated pathway of differential metabolites.

A total of 49 urine metabolites significantly changed after the exercise, including 23 upregulated and 26 downregulated metabolites (Figure 4a,b). Among the differential metabolites, some metabolites have been reported. For example, a significant increase in hypoxanthine was observed after the exercise in this study, and a similar phenomenon was observed after bicycle exercise [29] and marathon sports [30]. An increase in lactate is also a common phenomenon [23]. However, a few metabolites exhibited different trends between previous studies and this study. For example, inosine increased in this study but decreased by 34.79% after marathon sports [30], which may be due to different exercise prescriptions. In fact, most differential metabolites, especially those with a high fold change, have not been reported. Therefore, this study expanded the understanding of urine metabolite variation after high-intensity exercise.

The differential metabolites indicated the changes in metabolic pathways (Figure 4c,d). The tricarboxylic acid (TCA) cycle and pyruvate cycle, both of which are the most common energy metabolism pathways [31,32], were significantly enriched after the exercise and accounted for a higher energy supply during exercise. Upregulated glyoxylate and dicarboxylate metabolism, glycerolipid metabolism, and inositol phosphate metabolism are capable of generating the intermediate metabolites of the TCA cycle, supporting high-energy metabolism [33,34]. Some amino acid metabolisms, e.g., alanine, aspartate, glutamate, and histidine metabolisms, were also upregulated, which benefits in improving exercise performance [35,36]. Some metabolism pathways related to EIMD were also enriched. For example, alpha-linolenic acid (ALA) metabolism was downregulated, which was due to ALA simulating the generation of ROS [37]. Some antioxidant metabolisms, e.g., taurine, hypotaurine, ascorbate, and aldarate, were downregulated, which may be due to antioxidant consumption during exercise [38].

### 2.3. Potential Urinary Biomarker for EIMD Prediction

**Figure 5:**
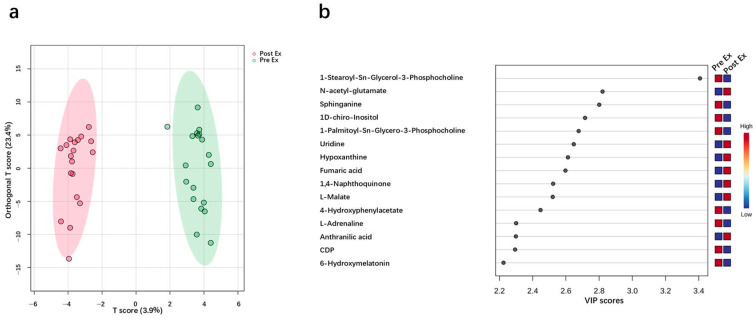
(**a**) Discriminant analysis with an OPLS-DA model; (**b**) the metabolites with high VIP values.

The discriminant analysis of 36 urine samples was performed using OPLS-DA. The 36 urine samples were clearly separated, which was in accord with the experiment groups (Figure 5a). Figure 5b lists the top 15 metabolites ranked by variable importance in the projection scores (VIP), including 1-Stearoyl-Sn-Glycerol-3-Phosphocholine, N-acetyl-glutamate, Sphinganine, 1D-chiro-Inositol, 1-Palmitoyl-Sn-Glycero-3-Phosphocholine, Uridine, Hypoxanthine, Fumaric acid, 1,4-Naphthoquinone, L-Malate, 4-Hydroxyphenylacetate, L-Adrenaline, Anthranilic acid, CDP, and 6-Hydroxymelatonin. All these metabolites have high Z-scores, ranging from 1.1 to 2.7. Differential metabolites with high VIP values or Z-scores are commonly used as potential biomarkers [39,40]. Thus far, these metabolites have never been reported in research on EIMD. Therefore, this study is the first to propose that these metabolites have great prospects for EIMD prediction.

**Figure 6:**
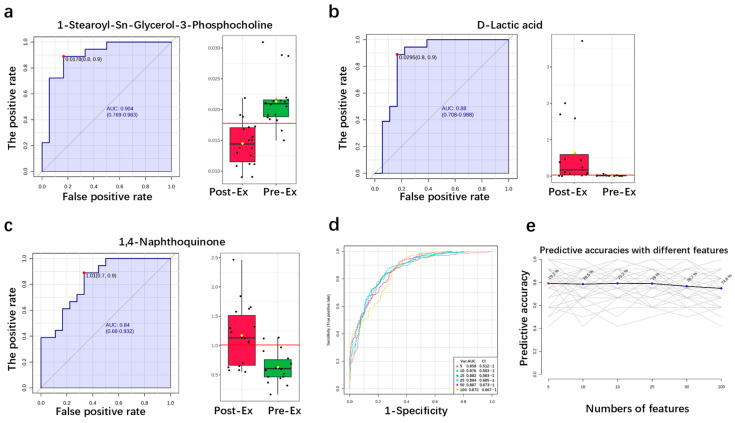
EIMD prediction with potential urinary biomarkers: (**a**–**c**) prediction performance of single urinary metabolite; (**d**) AUC of multiple metabolites; (**e**) predictive accuracy with an increase in metabolite number. (The red line represents a critical reference value for potential urinary biomarkers; The numerous thin gray lines represent individual predictive models trained on different subsets of features, and each line tracks how one specific model’s accuracy changes as the number of features increases from 5 to 100).

ROC curves were used to evaluate the prediction performance of these potential biomarkers (Figure 6a–c). According to the ranking of the area under the curve (AUC), the top three potential urinary biomarkers were 1-Stearoyl-Sn-Glycerol-3-Phosphocholine, D-Lactic acid, and 1,4-Naphthoquinone. Notably, all three potential urine metabolites exhibited high sensitivity and specificity for EIMD prediction. Among these metabolites, 1-Stearoyl-Sn-Glycerol-3-Phosphocholine exhibited the highest sensitivity of 88.9% and the highest specificity of 83.3%, with the highest AUC of 0.904. Furthermore, the prediction performance by using a single biomarker is enough because the combination of multiple biomarkers does not result in better EIMD prediction performance. As shown in Figure 6d, the AUC decreased to 0.858 when using five metabolites for EIMD prediction. Additionally, the predictive accuracy was less than 80% when using multiple biomarkers (Figure 6e). Therefore, it was concluded that a single urine biomarker shows great prospects in EIMD prediction, and the best biomarker is 1-Stearoyl-Sn-Glycerol-3-Phosphocholine.

Thus far, athletes mainly use the blood biochemical index or biomedical image to diagnose EIMD [28]. However, blood biochemical analyses are invasive, and biomedical image observation is hysteretic. Thus far, non-invasive methods for EIMD prediction have not been reported, with limited knowledge on EIMD biomarkers being available. In this study, the changes in urinary metabolites after EIMD occurrence have been revealed, and potential biomarkers for EIMD prediction were screened. Moreover, the potential biomarkers are small-molecule metabolites and could be rapidly quantified using molecular imprinting electrochemistry, immune-electrochemistry, or chemiluminescence techniques, which provide a basis for the development of portable and non-invasive methods for EIMD. Future studies will further investigate the practicality of the potential biomarkers in EIMD prediction and develop portable prediction equipment.

## 3. Materials and Methods

### 3.1. Subjects

In this study, 18 young male students were recruited as participants, with an age range of 21–24 and an average age of ~23. Table 1 shows the heights, weights, and BMIs of the participants. To mitigate the influence of gender differences, all participants were male. The questionnaire analysis confirmed that none of the participants had a regular exercise routine or had ever engaged in any training programs. Furthermore, none of the participants had cardiovascular diseases, renal disorders, or metabolic diseases by their self-reporting. All participants exhibited normal levels of the blood biochemical index of EIMD without exercise. All the participants provided written informed consent before exercise, and approval was obtained from the ethics committee of Beijing Institute of Technology [BIT-EC-H-2022143].

### 3.2. Exercise Protocol and Sample Collection

The exercise protocol for all participants was the same, including the rowing exercise followed by continuous squats. The rowing exercise was conducted using a rowing machine (MRH3208A, Mobifitness Co., Shanghai, China). The maximum resistance of the rowing machine was 32, and the resistance was set at 30 in this study. The training goal was to finish a total rowing distance of 3.0 km, which required approximately 600 strokes. The participants’ rowing posture was monitored and corrected in the training, with rapid pulling followed by a slow release. Multiple muscle groups were engaged in eccentric contraction during the slow release, which was expected to induce EIMD. Interval rests were set in the training, with 30 s of low-resistance rowing after 0.2 km of continuous high-resistance rowing. Moreover, the participants’ heart rates were monitored to maintain the predetermined intensity (approximately 85% maximum heart rate). Participants were asked to perform squats after rowing, and the count of consecutive squats was recorded. DOMS was evaluated by using a visual analog scale (VAS) at 24 h after exercise, in which 0 mm represents no pain while 100 mm represents extreme pain.

Blood and urine samples were collected at two time points: at rest (Pre-Ex) and immediately after exercise (Post-Ex). The samples collected were conducted on two adjacent days to avoid potential interference between blood sampling and training. The sampling was performed at approximately 10 AM, and the participants were asked to have the same breakfast at 8 AM. Having the same breakfast and sampling times aimed at avoiding dietary [41] and rhythm interference [24]. During the experiment, all participants were asked to abstain from alcohol, maintain a regular diet, and avoid overeating. Additionally, the participants were prohibited from engaging in any sports activities except for rowing training. Blood sampling was conducted at Beijing Institute of Technology Hospital and used for the blood biochemical index. Midstream urine samples were collected and used for the analysis of urinary metabolites.

### 3.3. Biochemical Index and Urine Metabolite Analyses

The biochemical indexes for EIMD analysis included CK, lactate dehydrogenase (LDH), and hydroxybutyrate dehydrogenase (HBDH). All biochemical index analyses were performed with the assistance of Beijing Di An Diagnostics Medical Laboratory by using enzyme-coupled reaction methods. Urine metabolites were analyzed using quasi-targeted metabolomics, which is characterized by high-throughput identification and relatively accurate quantification. A brief description of quasi-targeted metabolomics analysis is as follows. First, protein in the urine samples was removed using methanol, and the metabolites were extracted by freeze-drying. Then, the metabolites were analyzed using liquid chromatography–mass spectrometry. A triple quadrupole-linear ion trap mass spectrometer (QTRAP 6500+, AB Sciex Pte. Ltd., Framingham, MA, USA) was used. Finally, metabolites were accurately identified using the Novogene database and were relatively quantified by multiple reaction monitoring.

### 3.4. Bioinformatics Analysis

Urine metabolite concentrations were corrected to avoid the effects of urinary hydration status [42,43]. The correction was performed by calculating the ratio of the peak area of the metabolite to that of creatinine in the same sample. Bioinformatics analysis was performed after correction using the MetaboAnalyst 6.0 platform (www.metaboanalyst.ca (accessed on 10 February 2025)). Both principal component analysis (PCA) and clustering heatmaps were used to analyze the overall metabolite changes and trends. Pattern hunter was utilized to identify the urine metabolites that strongly changed related to the blood biochemical index. A paired t-test was used to identify significantly changed metabolites, and the Kyoto Encyclopedia of Genes and Genomes (KEGG) module was employed to analyze enriched metabolic pathways after exercise. Orthogonal partial least squares discriminant analysis (OPLS-DA) was applied to identify the metabolites that were capable of discriminating EIMD. The receiver operating characteristic curve (ROC) was used to screen EIMD urine biomarkers and analyze EIMD prediction performance.

## 4. Conclusions

High-intensity rowing exercise was seen to induce EIMD occurrence in participants who did not have regular exercise habits. The urinary metabolites obviously changed post-exercise. Most differential metabolites were newly reported and were related to energy production, exercise performance, and EIMD development. Potential urinary biomarkers for EIMD prediction were screened with high sensitivity and specificity, and a single biomarker exhibits better prediction performance than the combination of multiple metabolites. This study proposes a potential biomarker for EIMD prediction. Further validation should be carried out by using different exercise regimens. Despite the high correlation between metabolite changes and EIMD occurrence, the detailed mechanism of the correlation should be revealed. Furthermore, methods for rapid detection of these metabolites should be developed, e.g., molecular imprinting, bioelectrochemistry, or mini mass spectrometry. More efforts are needed to develop point-of-care testing equipment for EIMD prediction.

## Figures and Tables

**Table 1:** Basic characteristics of the subjects.

Physical Indicators	Range	Average Value
Height/cm	168–186	177.9
Weight/kg	66–86	72.1
BMI	20.3–27.1	22.8

## Data Availability

The metabolome data reported in this paper have been deposited in the OMIX, China National Center for Bioinformation/Beijing Institute of Genomics, Chinese Academy of Sciences (https://ngdc.cncb.ac.cn/omix/release/OMIX010439 (accessed on 7 Jun 2025)). Further inquiries can be directed to the corresponding author.

## Abbreviations

The following abbreviations are used in this manuscript:

EIMD	Exercise-induced muscle damage
DOMS	Delayed onset muscle soreness
CK	Creatine kinase
VAS	Visual analog scale
LDH	Lactate dehydrogenase
HBDH	Hydroxybutyrate dehydrogenase
PCA	Principal component analysis
KEGG	Kyoto Encyclopedia of Genes and Genomes
OPLS-DA	Orthogonal partial least squares discriminant analysis
ROC	Receiver operating characteristic
TCA	Tricarboxylic acid
ALA	alpha-Linolenic acid
VIP	Variable importance in the projection
AUC	Area under the curve

## References

[1] Analysts L., Amar D., Gay N.R., Jean-Beltran P.M., Generators L.D., Bae D., Dasari S., Dennis C., Evans C.R., MoTrPAC Study Group (2024). Temporal dynamics of the multi-omic response to endurance exercise training. Nature.

[2] Markus I., Constantini K., Hoffman J.R., Bartolomei S., Gepner Y. (2021). Exercise-induced muscle damage: Mechanism, assessment and nutritional factors to accelerate recovery. Eur. J. Appl. Physiol..

[3] Stozer A., Vodopivc P., Bombek L.K. (2020). Pathophysiology of Exercise-Induced Muscle Damage and Its Structural, Functional, Metabolic, and Clinical Consequences. Physiol. Res..

[4] Kim J., Lee J., Kim S., Ryu H.Y., Cha K.S., Sung D.J. (2016). Exercise-induced rhabdomyolysis mechanisms and prevention: A literature review. J. Sport Health Sci..

[5] Cleak M.J., Eston R.G. (1992). Muscle soreness, swelling, stiffness and strength loss after intense eccentric exercise. Br. J. Sports Med..

[6] Kelly R.S., Kelly M.P., Kelly P. (2020). Metabolomics, physical activity, exercise and health: A review of the current evidence. Biochim. Biophys. Acta-Mol. Basis Dis..

[7] Ebbeling C.B., Clarkson P.M. (1989). Exercise-induced muscle damage and adaptation. Sports Med..

[8] De Rosa A., Verrengia E.P., Merlo I., Rea F., Siciliano G., Corrao G., Prelle A. (2021). Muscle manifestations and CK levels in COVID infection: Results of a large cohort of patients inside a Pandemic COVID-19 Area. Acta Myol.

[9] Owens D.J., Twist C., Cobley J.N., Howatson G., Close G.L. (2019). Exercise-induced muscle damage: What is it, what causes it and what are the nutritional solutions?. Eur. J. Sport Sci..

[10] Neto G.R., Novaes J.S., Salerno V.P., Gonçalves M.M., Batista G.R., Cirilo-Sousa M.S. (2018). Does a resistance exercise session with continuous or intermittent blood flow restriction promote muscle damage and increase oxidative stress?. J. Sports Sci..

[11] Thorpe R., Sunderland C. (2012). Muscle damage, endocrine, and immune marker response to a soccer match. J. Strength Cond. Res..

[12] Lindsay A., Costello J.T. (2017). Realising the potential of urine and saliva as diagnostic tools in sport and exercise medicine. Sports Med..

[13] Fulford J., Eston R.G., Rowlands A.V., Davies R.C. (2015). Assessment of magnetic resonance techniques to measure muscle damage 24 h after eccentric exercise. Scand. J. Med. Sci. Sports.

[14] Martín-Fuentes I., Oliva-Lozano J.M., Muyor J.M. (2020). Electromyographic activity in deadlift exercise and its variants. A systematic review. PLoS ONE.

[15] Zhao J.J., Wang Y., Zhao D., Zhang L.Z., Chen P.J., Xu X. (2020). Integration of metabolomics and proteomics to reveal the metabolic characteristics of high-intensity interval training. Analyst.

[16] Sun T., Wu Y., Wu X.P., Ma H.F. (2017). Metabolomic profiles investigation on athletes’ urine 35 minutes after an 800-meter race. J. Sports Med. Phys. Fit..

[17] Pechlivanis A., Kostidis S., Saraslanidis P., Petridou A., Tsalis G., Mougios V., Gika H.G., Mikros E., Theodoridis G.A. (2010). ^1^H NMR-based metabonomic investigation of the effect of two different exercise sessions on the metabolic fingerprint of human urine. J. Proteome Res..

[18] Prado E., Souza G., Pegurier M., Vieira C., Lima-Neto A.B.M., Assis M., Guedes M.I.F., Koblitz M.G.B., Ferreira M.S.L., Macedo A.F. (2017). Non-targeted sportomics analyses by mass spectrometry to understand exercise-induced metabolic stress in soccer players. Int. J. Mass Spectrom..

[19] Ali A.M., Burleigh M., Daskalaki E., Zhang T., Easton C., Watson D.G. (2016). Metabolomic Profiling of Submaximal Exercise at a Standardised Relative Intensity in Healthy Adults. Metabolites.

[20] Mukherjee K., Edgett B.A., Burrows H.W., Castro C., Griffin J.L., Schwertani A.G., Gurd B.J., Funk C.D. (2014). Whole blood transcriptomics and urinary metabolomics to define adaptive biochemical pathways of high-intensity exercise in 50–60 year old masters athletes. PLoS ONE.

[21] Schader J.F., Haid M., Cecil A., Schoenfeld J., Halle M., Pfeufer A., Prehn C., Adamski J., Nieman D.C., Scherr J. (2020). Metabolite shifts induced by marathon race competition differ between athletes based on level of fitness and performance: A substudy of the Enzy-MagIC Study. Metabolites.

[22] Wang F.Q., Han J., He Q., Geng Z.F., Deng Z.W., Qiao D.C. (2015). Applying ^1^H NMR spectroscopy to detect changes in the urinary metabolite levels of Chinese half-pipe snowboarders after different exercises. J. Anal. Methods Chem..

[23] Jang H.-J., Lee J.D., Jeon H.-S., Kim A.-R., Kim S., Lee H.-S., Kim K.-B. (2018). Metabolic profiling of eccentric exercise-induced muscle damage in human urine. Toxicol. Res..

[24] Hammouda O., Chtourou H., Chahed H., Ferchichi S., Kallel C., Miled A., Chamari K., Souissi N. (2011). Diurnal variations of plasma homocysteine, total antioxidant status, and biological markers of muscle injury during repeated sprint: Effect on performance and muscle fatigue—A pilot study. Chronobiol. Int..

[25] Pokora I., Kempa K., Chrapusta S.J., Langfort J. (2014). Effects of downhill and uphill exercises of equivalent submaximal intensities on selected blood cytokine levels and blood creatine kinase activity. Biol. Sport.

[26] Gomes J.H., Mendes R.R., Franca C.S., Da Silva-Grigoletto M.E., Pereira da Silva D.R., Antoniolli A.R., de Oliveira E.S.A.M., Quintans-Júnior L.J. (2020). Acute leucocyte, muscle damage, and stress marker responses to high-intensity functional training. PLoS ONE.

[27] Cheng A.J., Jude B., Lanner J.T. (2020). Intramuscular mechanisms of overtraining. Redox Biol..

[28] Burt D., Hayman O., Forsyth J., Doma K., Twist C. (2020). Monitoring indices of exercise-induced muscle damage and recovery in male field hockey: Is it time to retire creatine kinase?. Sci. Sports.

[29] Berton R., Conceiçao M.S., Libardi C.A., Canevarolo R.R., Gáspari A.F., Chacon-Mikahil M.P.T., Zeri A.C., Cavaglieri C.R. (2017). Metabolic time-course response after resistance exercise: A metabolomics approach. J. Sports Sci..

[30] Shi R.F., Zhang J., Fang B.Q., Tian X.Y., Feng Y., Cheng Z.P., Fu Z.Y., Zhang J.J., Wu J.X. (2020). Runners’ metabolomic changes following marathon. Nutr. Metab..

[31] Su Y.B., Peng B., Li H., Cheng Z.X., Zhang T.T., Zhu J.X., Li D., Li M.Y., Ye J.Z., Du C.C. (2018). Pyruvate cycle increases aminoglycoside efficacy and provides respiratory energy in bacteria. Proc. Natl. Acad. Sci. USA.

[32] Pachnis P., Wu Z., Faubert B., Tasdogan A., Gu W., Shelton S., Solmonson A., Rao A.D., Kaushik A.K., Rogers T.J. (2022). In vivo isotope tracing reveals a requirement for the electron transport chain in glucose and glutamine metabolism by tumors. Sci. Adv..

[33] Sklirou E., Alodaib A.N., Dobrowolski S.F., Mohsen A.A., Vockley J. (2020). Physiological perspectives on the use of triheptanoin as anaplerotic therapy for long chain fatty acid oxidation disorders. Front. Genet..

[34] Lee J.-H., Jung I.-R., Tu-Sekine B., Jin S., Anokye-Danso F., Ahima R.S., Kim S.F. (2024). IPMK Deficiency Reduces Skeletal Muscle Oxidative Metabolism and Exercise Capacity. bioRxiv.

[35] Turgut M., Cinar V., Pala R., Tuzcu M., Orhan C., Telceken H., Sahin N., Deeh P.B.D., Komorowski J.R., Sahin K. (2018). Biotin and chromium histidinate improve glucose metabolism and proteins expression levels of IRS-1, PPAR-γ, and NF-κB in exercise-trained rats. J. Int. Soc. Sports Nutr..

[36] Nassis G.P., Sporer B., Stathis C.G. (2017). β-alanine efficacy for sports performance improvement: From science to practice. Br. J. Sports Med..

[37] Monaco C.M.F., Proudfoot R., Miotto P.M., Herbst E.A.F., MacPherson R.E.K., Holloway G.P. (2018). α-linolenic acid supplementation prevents exercise-induced improvements in white adipose tissue mitochondrial bioenergetics and whole-body glucose homeostasis in obese Zucker rats. Diabetologia.

[38] Seidel U., Huebbe P., Rimbach G. (2018). Taurine: A Regulator of Cellular Redox Homeostasis and Skeletal Muscle Function. Mol. Nutr. Food Res..

[39] Peng B., Su Y.B., Li H., Han Y., Guo C., Tian Y.M., Peng X.X. (2015). Exogenous alanine and/or glucose plus kanamycin kills antibiotic-resistant bacteria. Cell Metab..

[40] Zhao X.L., Chen Z.G., Yang T.C., Jiang M., Wang J., Cheng Z.X., Yang M.J., Zhu J.X., Zhang T.T., Li H. (2021). Glutamine promotes antibiotic uptake to kill multidrug-resistant uropathogenic bacteria. Sci. Transl. Med..

[41] Krug S., Kastenmüller G., Stückler F., Rist M.J., Skurk T., Sailer M., Raffler J., Römisch-Margl W., Adamski J., Prehn C. (2012). The dynamic range of the human metabolome revealed by challenges. Faseb J..

[42] Ma S.R., Lieberman S., Turino G.M., Lin Y.Y. (2003). The detection and quantitation of free desmosine and isodesmosine in human urine and their peptide-bound forms in sputum. Proc. Natl. Acad. Sci. USA.

[43] Lindsay A., Janmale T., Draper N., Gieseg S.P. (2014). Measurement of changes in urinary neopterin and total neopterin in body builders using SCX HPLC. Pteridines.

